# Preparation and Characterization of Chitosan-Coated Pectin Aerogels: *Curcumin* Case Study

**DOI:** 10.3390/molecules25051187

**Published:** 2020-03-06

**Authors:** Milica Pantić, Gabrijela Horvat, Željko Knez, Zoran Novak

**Affiliations:** Faculty of Chemistry and Chemical Engineering, University of Maribor, Smetanova 17, 2000 Maribor, Slovenia; milica.pantic1@um.si (M.P.); gabrijela.horvat@um.si (G.H.); zeljko.knez@um.si (Ž.K.)

**Keywords:** curcumin, pectin aerogels, chitosan coating, burst release, controlled release

## Abstract

The following study describes the preparation of pectin aerogels and pectin aerogels coated with an external layer of chitosan. For the preparation of chitosan-coated pectin aerogels, a modified coating procedure was employed. Since pectin as well as pectin aerogels are highly water soluble, a function of chitosan coating is to slow down the dissolution of pectin and consequently the release of the active substances. Textural properties, surface morphologies, thermal properties, and functional groups of prepared aerogels were determined. Results indicated that the coating procedure affected the textural properties of pectin aerogels, resulting in smaller specific surface areas of 276 m^2^/g, compared to 441 m^2^/g. However, chitosan-coated pectin aerogels still retained favorable properties for carriers of active substances. The case study for prepared aerogels was conducted with curcumin. Prior to in-vitro release studies, swelling studies were performed. Curcumin’s dissolution from both aerogels showed to be successful. Pectin aerogels released curcumin in 3 h showing a burst release profile. Chitosan-coated pectin aerogels prolonged curcumin release up to 24 h, thus showing a controlled release profile.

## 1. Introduction

Polysaccharides have gained increasing attention in biomedicine within the last decade. Due to their exquisite properties such as non-toxicity, biodegradability, and biocompatibility, they are listed as excellent candidates for use in various biomedical formulations.

Pectin is a linear polysaccharide mainly consisting of galacturonic acid units which are connected via α-(1-4) bonds. Gelling properties of pectin depend on the ratio of esterified and amidated acid groups, and particularly the type of pectin (low methoxyl pectin, high methoxyl pectin, or amidated pectin) [1]. Different gelation methods have been reported in the literature: ionotropic gelation with Ca^2+^ [2], ionotropic gelation with ethanolic solution of Ca^2+^ [3], emulsification in oil followed by coagulation in ethanol [4], and ethanol gelation [5]. Pectin aerogels are highly water soluble in the same way as pectin. They could be used for improving the dissolution and bioavailability of poorly water-soluble drugs [6].

Chitosan is a linear polysaccharide consisting of linked β-(1-4)-glucosamine units. It can be obtained by deacetylation of chitin from seafood industry waste, such as squid pens and crab shells. Gels can be formed either by irreversible covalent links with chemical cross-linkers, such as glutaraldehyde, or by various reversible links with ions and polyelectrolyte complexes. The latter are formed by dissolution of chitosan in an acidic medium, followed by precipitation in an alkaline solution, which is the simplest way of preparing chitosan gels [7]. Chitosan aerogels, in the literature usually referred to as scaffolds, are in most cases used for tissue engineering applications [8]. Unlike pectin, chitosan is soluble in an acidic medium, in lower pH ranges. This property is very important and opens up possibilities for new applications.

The drug release properties from water-soluble polysaccharides, such as pectin, suffer some problems. The main disadvantage is the burst release of active substances due to quick breakdown of gels in vitro. To improve the performance of water-soluble polysaccharides, chitosan coatings can be added to protect the core and prolong the release of active substances trapped inside. There are efforts in the literature to achieve these formulations and mentioned effect [9,10,11,12,13].

Curcumin is a yellow pigment present in the spice turmeric (*Curcuma longa*). It is associated with antioxidant, anti-inflammatory, anticancer, antiviral, and antibacterial activities, confirmed by more than 6000 citations and hundreds of clinical studies [14]. Curcumin, however, has poor absorption, biodistribution, metabolism, and bioavailability. There are suggestions to overcome these problems, by incorporating curcumin into formulations such as nanoparticles, liposomes, micelles, and phospholipid complexes [15,16,17,18].

The aim of this study was to improve the bioavailability of curcumin by attaching it to highly water-soluble pectin aerogels in the first step. Secondly, the curcumin release was optimized by adding a chitosan layer over the pectin core.

To the best of our knowledge, this is one of the first studies on the topic of chitosan coatings over pectin aerogels.

## 2. Results

### 2.1. Synthesis of Pectin Aerogels and Chitosan-Coated Pectin Aerogels

**Figure 1:**
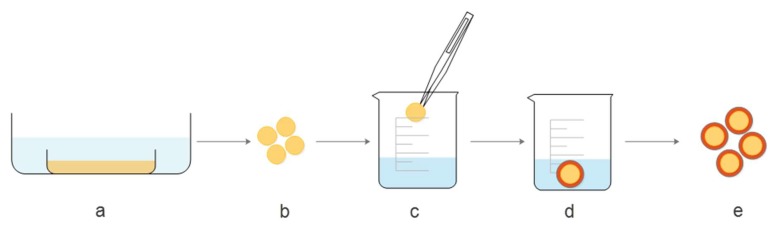
Schematic presentation of the coating procedure.

Figure 1 shows a schematic presentation of the modified coating method, applied for covering the pectin core with a chitosan layer. In the first step (a), 4% (*w*/*w*) pectin solution was prepared, poured into molds and covered with ethanol to obtain gels. Once the stable gel was obtained, tablet shape forms were cut (b) for further treatment. Step (c) refers to dipping pectin cores into 1.5% (*w*/*w*) chitosan solution in 0.2 M CH_3_COOH. To attach chitosan to the pectin core, tablets were transferred to NaOH solution in ethanol (d), which triggered the gelation of chitosan. In the last step, before further processing, pectin gels coated with chitosan were washed out with ethanol (e).

**Figure 2:**
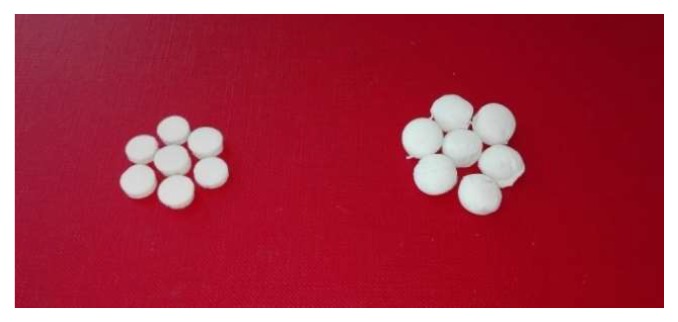
Pectin aerogels and chitosan-coated pectin aerogels.

To prepare aerogels from gels, a supercritical drying technique was applied at 120 bar and 40 °C for 6 h. The aerogels obtained are presented in Figure 2. Chitosan-coated pectin aerogels (right side) are more massive with clearly changed shapes. Unlike the pectin aerogels (left side), which have a precise, tablet-shaped form, chitosan-coated pectin aerogels have less defined shapes. The average diameter-to-height ratio for pectin aerogels is 3, while for chitosan-coated pectin aerogels, it is 1.5.

### 2.2. Characterization of Pectin Aerogels and Chitosan-Coated Pectin Aerogels

#### 2.2.1. Textural properties

Specific surface areas were measured using N_2_ adsorption–desorption analysis. Furthermore, bulk and skeletal densities were determined, and porosity was calculated. All values are shown in Table 1.

Specific surface area of chitosan-coated pectin aerogels is 276 m^2^/g, which is significantly lower compared to pure pectin aerogels, which have a surface area of 441 m^2^/g. It seems that the coating procedure affected the structure of pure pectin aerogels, resulting in smaller specific surface areas. However, the specific surface area of 276 m^2^/g is still high enough for loading of the active substances (such as curcumin) and the potential as a drug carrier. Very high porosities of 96 ± 0.05% and 94.8 ± 0.03% undoubtedly shows aerogels nature of both pectin and chitosan-coated pectin materials.

**Figure 3:**
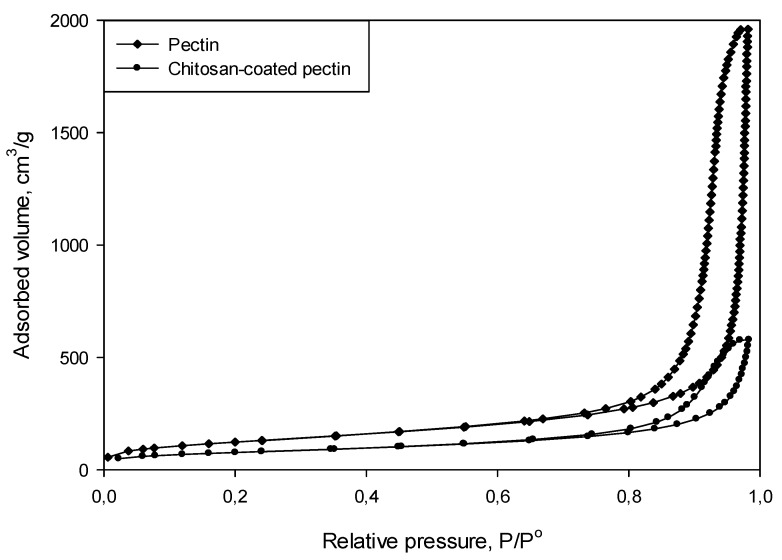
Adsorption–desorption isotherms for pectin aerogel and chitosan-coated pectin aerogels.

The N_2_ adsorption–desorption isotherms for both pectin aerogels and chitosan-coated pectin aerogels, presented in Figure 3 could be classified as type IV isotherms. From the types of isotherms, it can be concluded that the prepared aerogels are mesoporous materials.

#### 2.2.2. Scanning Electron Microscopy

**Figure 4:**
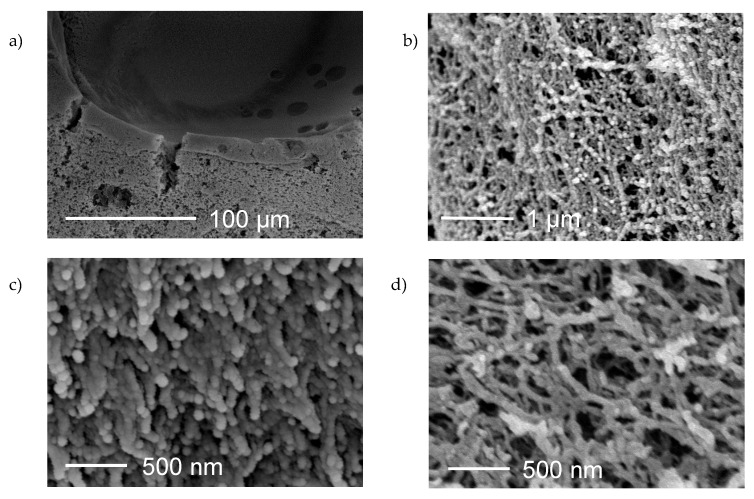
Scanning electron microscopy (SEM) images: (**a**) chitosan-coated pectin aerogels, the inner part (100 µm scale); (**b**) chitosan-coated pectin aerogels, the outer part (1 µm scale); (**c**) pectin aerogel (500 nm scale); and (**d**) chitosan-coated pectin aerogels (500 nm scale).

Scanning electron microscopy (SEM) was employed for determining the surface morphology of prepared aerogels. The coating of pectin core with chitosan layer was confirmed by SEM image on a 100 µm scale. Figure 4a undoubtedly shows a two-layer aerogel. The upper layer represents a chitosan coating over a pectin core. Porous structure is visible in both layers. Furthermore, Figure 4b represents the outer part of the coating on a 1 µm scale, confirming the porous structure of the chitosan.

Figure 4c,d present the pore network of pectin aerogels and chitosan-coated pectin aerogels on a 500 nm scale. In both cases, the structure is highly porous, having a complex interconnected network of pores. The structure of pectin aerogels seems to be more compact compared to the structure of chitosan-coated pectin aerogels, which is expected since the specific surface area and porosity of pure pectin aerogels is higher.

#### 2.2.3. Thermal Analysis

Thermogravimetry (TGA) and differential scanning calorimetry (DSC) were carried out simultaneously. The analyses were performed at air atmosphere at a temperature range from 30 to 600 °C, with a heating rate of 10 °C per minute.

**Figure 5:**
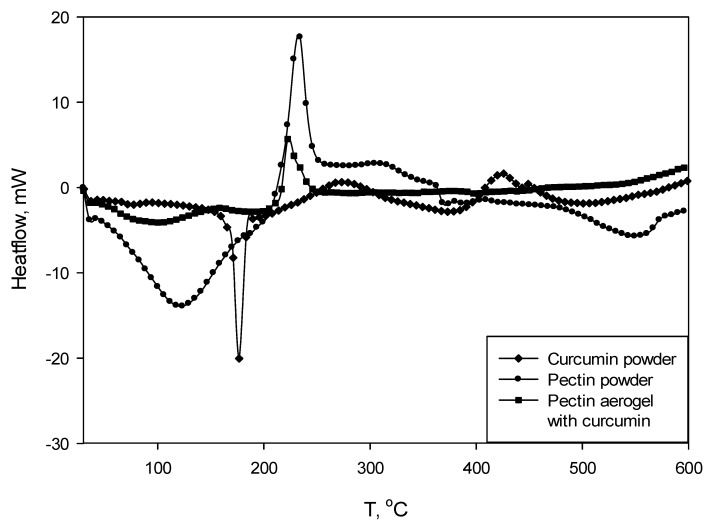
Differential scanning calorimetry (DSC) curves for curcumin powder, pectin powder and pectin aerogel loaded with curcumin.

Figure 5 shows DSC curves for pectin polysaccharide (powder), curcumin (powder), and pectin aerogel loaded with curcumin. Chitosan-coated pectin aerogels loaded with curcumin were not subjected to TGA/DSC analysis due to the size and weight limits.

The DSC curve for curcumin shows a clear peak at 178 °C, indicating the melting point of the substance. The curve for pectin shows an exothermic peak at 237 °C, indicating a degradation of this polysaccharide. As for pectin, the DSC curve for pectin aerogel loaded with curcumin has an exothermic degradation peak at 225 °C. In this case, the peak is shifted to the left, which means that the degradation occurs earlier. On the other hand, the melting peak of the curcumin is not visible on the pectin–curcumin curve.

**Figure 6:**
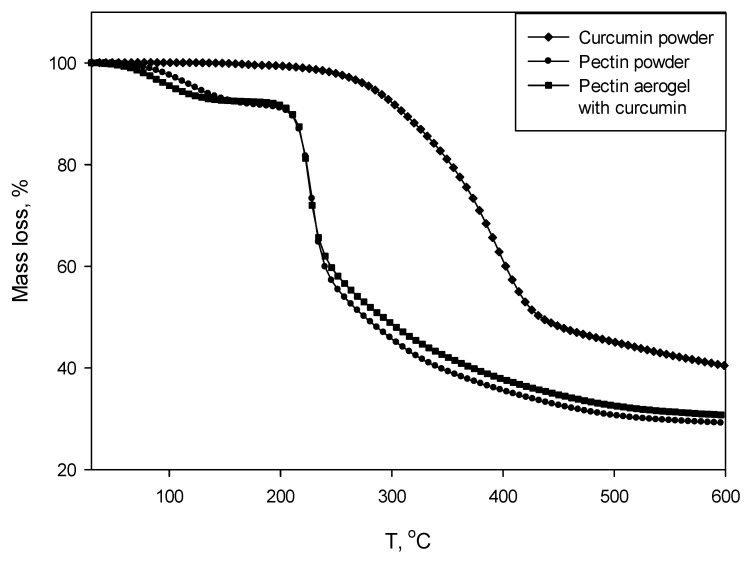
Thermogravimetry (TGA) curves for curcumin powder, pectin powder, and pectin aerogel loaded with curcumin.

Simultaneously with DSC, TGA was measured as presented in Figure 6.

The values read from TGA curves are given in Table 2. The thermal degradation of pectin polysaccharide as well pectin aerogel loaded with curcumin occurs in the two steps. The main decomposition (62%) occurs at higher temperatures, which is confirmed by DSC curves (exothermic degradation peaks at 237 and 225 °C). Decomposition of curcumin of 59% occurs in one step and corresponds to the melting of the substance.

#### 2.2.4. Fourier Transform Infrared Spectroscopy

**Figure 7:**
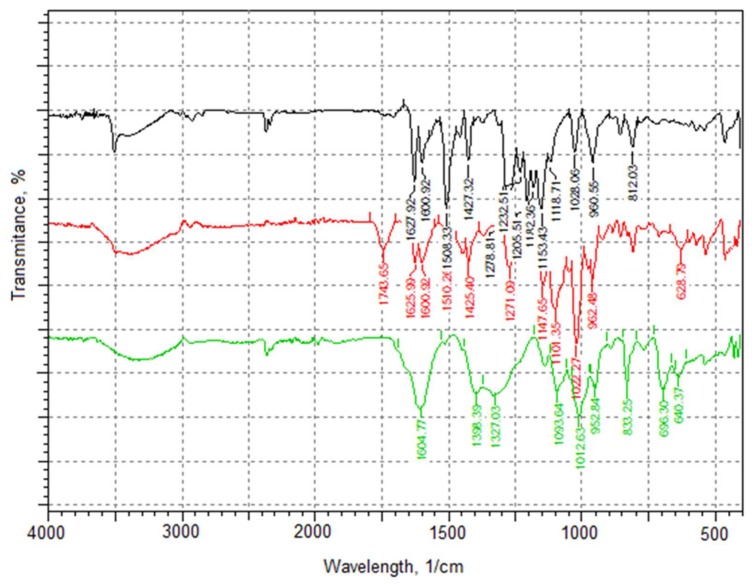
Infrared (IR) spectra: curcumin powder (black), pectin aerogel loaded with curcumin (red), and chitosan-coated pectin aerogel loaded with curcumin (green).

Figure 7 presents infrared (IR) spectra for curcumin powder, pectin aerogels loaded with curcumin, and chitosan-coated pectin aerogels also loaded with curcumin.

IR spectrum of curcumin clearly shows characteristic peaks [19]: 3500 cm^−1^ for phenolic O-H stretching, 1628 cm^−1^ aromatic moiety C=C stretching, and 1600 cm^−1^ benzene ring stretching vibrations identifying curcumin. Furthermore, the IR spectrum contains C=O and C=C vibrations at 1508 cm^−1^, olefinic C-H bending vibrations at 1427 cm^−1^, and aromatic C-O stretching vibrations at 1278 cm^−1^.

The IR spectrum for pectin aerogels loaded with curcumin shows a characteristic peak at 1743 cm^−1^, presenting esterified carboxyl groups. Peaks at 1625 cm^−1^ and 1600 cm^−1^ are clearly visible, confirming the presence of loaded curcumin.

Lastly, the IR spectrum of chitosan-coated pectin aerogels show characteristic peaks at 1604 cm^−1^ presenting N-H bending of the primary amine, 1398 cm^−1^ and 1327 cm^−1^ presenting CH_2_ bending and CH_3_ symmetrical deformations. These peaks are the characteristic signature of chitosan. Characteristic peaks for curcumin, however, are overlapping with peaks of chitosan; hence, they are not visible in the spectrum. The presence of curcumin was later confirmed by in-vitro release studies.

### 2.3. Swelling Studies

Swelling studies are more or less able to predict the behavior of aerogels during in-vitro release studies. The studies were performed for both pectin aerogels and chitosan-coated pectin aerogels without the presence of curcumin. Experiments were performed in simulated gastric fluid (SGF) at pH = 1.2, and simulated intestinal fluid (SIF) at pH = 6.8, in both cases for 24 h. The aerogels were afterwards compared to see the influence of the coating on their behavior in SGF/SIF.

**Figure 8:**
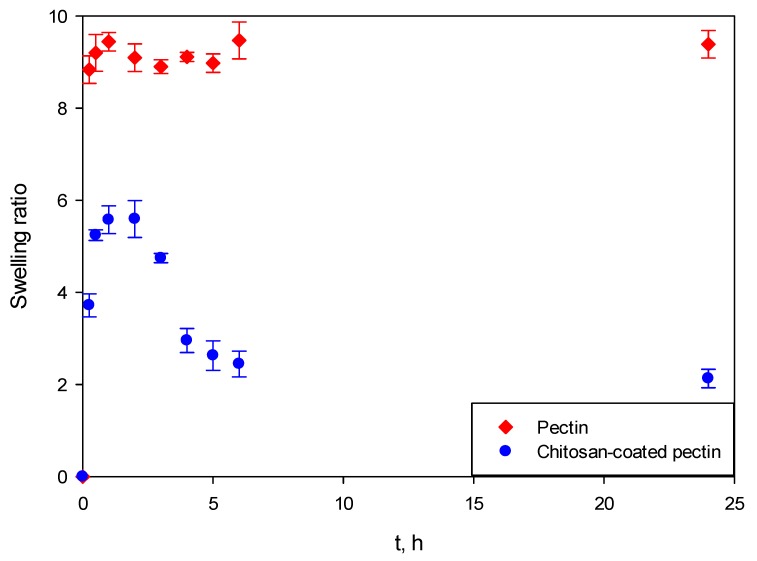
Swelling behavior of pectin aerogel and chitosan-coated pectin aerogel in SGF at pH = 1.2 for 24 h.

Figure 8 presents the swelling behavior of both aerogels at SGF. It can be clearly seen that pectin aerogels and chitosan-coated pectin aerogels behave completely differently.

Both pectin aerogels as well as chitosan-coated pectin aerogels in contact with SGF immediately swelled. However, once pectin aerogels reached their maximal swelling ratio, they stayed unchanged for the next 24 h. This means that they are stable in the mentioned fluid. Chitosan-coated pectin aerogels reached their maximal swelling ratio after 2 h. Unlike pectin, they started to decompose after 2 h and continued decomposing over the next 4 h. Chitosan-coated pectin aerogels have an additional chitosan layer which is soluble at a lower pH. This means that the chitosan layer swells and afterwards decomposes. Once the chitosan layer decomposes after approximately 6 h, the pectin core stays stable in the SGF, in the same way as pectin aerogels.

**Figure 9:**
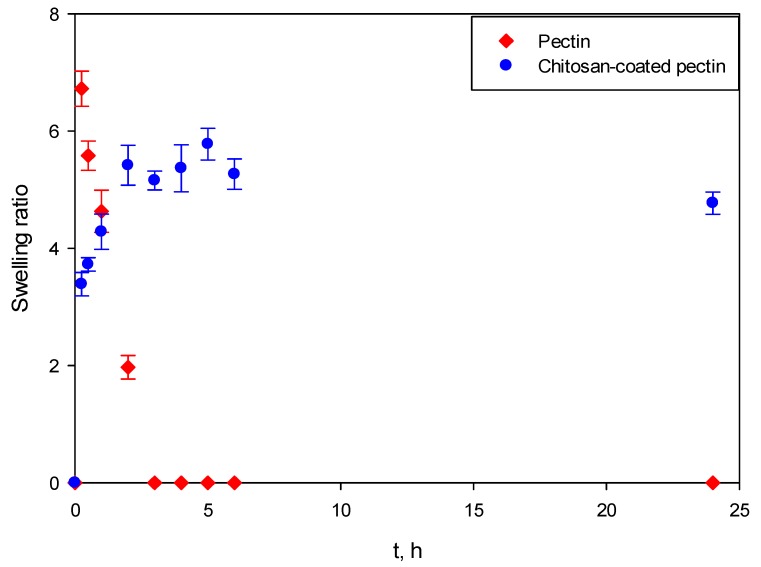
Swelling behavior of pectin aerogel and chitosan-coated pectin aerogels in SIF at pH = 6.8 for 24 h.

In SIF, the behavior of aerogels again differs, as shown in Figure 9. Almost immediately after contact with SIF, pectin aerogels reach their maximal ratio. After 3 h, pectin aerogels completely decompose. On the other hand, the swelling ratio of chitosan-coated pectin aerogels increases slowly, reaching its maximal ratio after approximately 5 h. Afterwards, they stay stable in the mentioned fluid. In this case, a chitosan layer over the pectin core prolongs the decomposition of the pectin core from 3 to 6 h. Once the pectin core is decomposed, the chitosan layer is stable in SIF, in the same way pectin is in SGF.

It is important to emphasize that swelling studies in SGF and SIF were performed independently. Entirely new aerogels were used for both SGF and SIF. The human body, however, functions differently. Once a drug is taken, it stays in the stomach up to 2 h, and then moves (if it does not dissolve) to the intestines for another 6 to 24 h, depending on bowel movements and their emptying.

Based on the aforementioned mechanism, drug release studies for both pectin aerogels and chitosan-coated pectin aerogels were performed first in SGF for 2 h and were afterwards transferred to SIF for an additional 22 h.

### 2.4. In-Vitro Curcumin Release Studies from Aerogels

To increase the bioavailability of curcumin, two types of aerogels were proposed as carriers: pectin aerogels and pectin aerogels coated with a layer of chitosan. The release of curcumin was tested through in-vitro release studies. Additionally, dissolution of curcumin powder was performed as a comparison.

**Figure 10:**
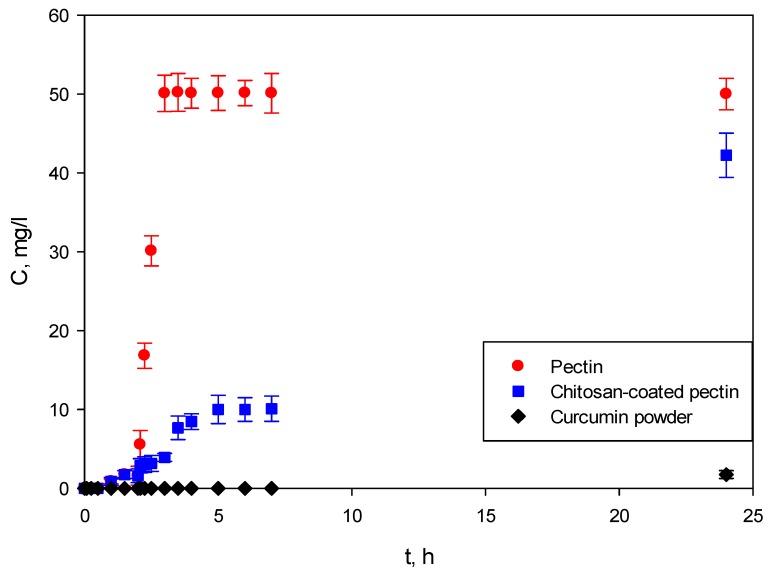
In-vitro curcumin release studies: 2 h in SGF followed by 22 h in SIF, 24 h overall.

As shown in Figure 10, the studies were performed for the first 2 h in SGF at pH = 1.2, and SIF at pH = 6.8 for the next 22 h. Weighted samples were immersed in SGF, after which they were immediately transferred to SIF. The experiments were monitored for 24 h.

In the first part of the study, both pectin aerogels and chitosan-coated pectin aerogels showed almost no release of curcumin in SGF at pH = 1.2. Both aerogels swell in this environment, but apparently not enough to release a significant amount of curcumin. These results are in good agreement with swelling studies, in which it was shown that both aerogels needed approximately 2 h to reach their maximal swelling ratio. In this case, this behavior of aerogels is highly desirable since the absorption of curcumin occurs in the intestine [14]; hence, there is no need for its release in the stomach.

When transferred to SIF, the release of curcumin from pectin aerogels is completely different from that of -chitosan-coated pectin aerogels. Pectin aerogels release all of their curcumin after just 1 h in SIF, of 3 h overall.

If the desired release is controlled release, which it is in most cases, then the release from pectin aerogels has to be slowed down, but not too much. Controlling drug diffusion from the dosage form is an excellent approach to maintaining therapeutic levels of the drug in the body. Controlled drug release in this particular case is important because of the poor adsorption of curcumin by the human body. It is crucial that a controlled amount of the drug reaches the body so that as much as possible can be adsorbed. If a large amount of the drug reaches the body at once, much of it will pass through without any adsorption at all.

Chitosan-coated pectin aerogels demonstrated slower release of curcumin, prolonged up to 24 h. As seen from Figure 10, curcumin was released for 24 h, leaving enough time for the substance to be adsorbed and metabolized by the body.

Lastly, curcumin powder was shown to be practically insoluble in body fluids, showing almost no dissolution after 24 h.

## 3. Discussion

Besides pure pectin aerogels, chitosan-coated pectin aerogels were prepared. The shape of latter slightly changed, resulting in a more massive appearance and less defined shapes.

N_2_ adsorption–desorption analysis showed that chitosan-coated pectin aerogels had significantly reduced specific surface areas and porosity compared to pure pectin aerogels. Namely, the preparation procedure affected the pore network, reducing the specific surface areas of pure pectin aerogels from 441 m^2^/g to 276 m^2^/g. Transferring pectin cores to the NaOH solution apparently caused the shrinkage of pectin cores and some damages in the pore network. The adsorption capacity of pectin aerogels is higher compared to the adsorption capacity of chitosan-coated pectin aerogels, based on adsorption–desorption isotherms.

Scanning electron microscopy revealed porous structures for both pure pectin aerogels and chitosan-coated pectin aerogels, in both pectin core and chitosan layer. However, the structure of pectin aerogels showed to be more compact. SEM images are in good agreement with N_2_ adsorption–desorption analysis, since pectin aerogels have higher specific surface areas. SEM images of chitosan-coated pectin aerogels show less compact structure, caused by the coating procedure and some damages to the pore network due to the shrinkage.

Thermal analysis consisted out of simultaneous thermogravimetry and differential scanning calorimetry to obtain TGA and DSC curves. The DSC curve of pectin aerogel loaded with curcumin compared to pectin polysaccharide showed a shifted peak, indicating the earlier degradation. A possible cause is the presence of curcumin. However, the melting peak of the curcumin is not visible on the pectin–curcumin curve. The overall mass of analyzed pectin aerogel loaded with curcumin is approximately 10 mg. This means that the mass of curcumin is quite low and could be simply covered or not detected in this case. TGA curves of pectin polysaccharide and pectin aerogels loaded with curcumin showed that the thermal degradation occurs in two steps.

Determined IR spectrum of pectin aerogels loaded with curcumin confirmed the presence of curcumin. The chemical structure of pectin, however, was not changed. This was verified by characteristic peaks for pectin that are still present in the spectrum. This means that the pectin aerogel serves as carrier, without chemical changes in the structure caused by the presence of active substances. In the case of chitosan-coated pectin aerogels, the characteristic peaks for curcumin are overlapping with characteristic peaks for chitosan. Even though it is not visible in the spectrum, the presence of curcumin was confirmed by further in-vitro release studies. In this case as well, the characteristic peaks for chitosan are present, again proving preserved chemical structure of polysaccharide.

Behavior of unloaded aerogels was tested in SGF at pH = 1.2 and SIF at pH = 6.8. At SGF, pectin aerogels showed to be stable. Contrary, chitosan-coated pectin aerogels started their decomposition after 2 h and finished the decomposition after 4 h. Actually, only the coating made of chitosan decomposed since the chitosan is soluble in acidic medium. Pectin core was, however, stable. This means that by using chitosan coating, pectin core is protected from the decomposition. Behavior in SIF fluids completely differs. While pectin aerogels completely decomposed after 3 h, chitosan-coated pectin aerogels are stable in neutral fluid. Chitosan coating was able to slow down and prolong the decomposition of pectin from 3 h up to 6 h. This behavior opens up the possibility for retaining the drug for a longer time period inside the core and, later on, the drug’s retardation during release.

Release of curcumin from both pure pectin aerogels and chitosan-coated pectin aerogels was tested through in-vitro studies and compared with the dissolution of curcumin powder.

Release of curcumin in SGF was retained for both aerogels. However, when transferred to SIF, pectin aerogels show burst release within just 1 h (3 h overall). This result is in good agreement with the swelling studies, in which pectin aerogels were completely decomposed after just 3 h spent in SIF. During drug release studies, pectin aerogels swelled in SGF and decomposed and released curcumin after 1 h in SIF. The dissolution and bioavailability of curcumin is tremendously improved, compared to standard curcumin. The porous network structure of aerogels enabled the surrounding of the molecules of curcumin by molecules of water, thus providing the possibility of faster dissolution. Even though the dissolution of curcumin was significantly improved, the release was still a burst. In the case of chitosan-coated pectin aerogels, release of curcumin was prolonged up to 24 h. By covering pectin with a chitosan layer, the core and, consequently, the curcumin trapped inside are partially protected. This formulation slowly swells, and consequently slowly releases curcumin. By protecting the highly soluble pectin core with a chitosan layer, controlled release of curcumin was achieved. As expected, curcumin powder showed almost no dissolution for the tested period.

## 4. Materials and Methods

### 4.1. Materials

Polysaccharides, pectin from citrus (TCI Europe), and chitosan (Sigma Aldrich, medium molecular weight) were used for the synthesis of pectin aerogels and pectin aerogels coated with a layer of chitosan. Ethanol absolute, C_2_H_5_OH (Merck, Darmstadt, Germany) and carbon dioxide, CO_2_ ( purity 99.5%, Messer, Ruše, Slovenia) were introduced for the solvent exchange during synthesis of gels and supercritical drying of prepared gels, respectively. Curcumin (purity ≥ 65%, Merck, Darmstadt, Germany) was used as an active substance for prepared aerogels. Chitosan was dissolved in acetic acid, CH_3_COOH (purity ≥ 98%, Fisher Scientific, Pittsburgh, PA, USA). Hydrochloric acid, HCl (purity 37%, Merck, Darmstadt, Germany), potassium phosphate monobasic, KH_2_PO_4_ (purity ≥ 98%, Merck, Darmstadt, Germany) and sodium hydroxide, NaOH (purity ≥ 98%, Merck, Darmstadt, Germany) were employed for the preparation of simulated gastric (SGF) and simulated intestinal fluid (SIF) for swelling and drug release studies. SGF (HCl) with pH = 1.2 was prepared by diluting 8.3 mL of 37% HCl to 1000 mL with miliQ water. SIF (phosphate buffer solution) with pH = 6.8 was prepared by mixing 250 mL of 0.2 M KH_2_PO_4_ and 112 mL of 0.2 M NaOH and diluted to 1000 mL with miliQ water.

### 4.2. Methods

#### 4.2.1. Synthesis of Pectin Aerogels

Pectin aerogels were prepared by ethanol-induced gelation, a method designed in our laboratory [20]. A weighed amount of pectin was dissolved in miliQ water to obtain a 4% (*w*/*w*) solution. The solution was strengthened by addition of a known amount of ethanol (not more than 10%). The hardened solution was transferred into molds and soaked in ethanol to obtain gels. The gels were cut into tablet form using a precise cutter with a diameter of 12.5 mm.

#### 4.2.2. Synthesis of Chitosan-Coated Pectin Aerogels

For the preparation of pectin aerogels coated with a chitosan layer, a coating procedure was designed. Pectin core gels were prepared as described above. Chitosan solution, 1.5% (*w*/*w*), was prepared by dissolving a known amount of chitosan into 0.2 M CH_3_COOH. Pectin core gels were soaked in a chitosan solution to bring the solution over the core. To attach the chitosan layer, soaked pectin cores were immediately transferred into 2 M NaOH. 2 M NaOH was prepared in ethanol. As pectin cores covered with chitosan solution were transferred into the NaOH solution, they immediately attached, since NaOH triggers the gelation of chitosan. After 20 min, the samples were transferred into ethanol to wash out the remaining NaOH.

#### 4.2.3. Loading of Curcumin

The addition of curcumin was conducted using ethanol during the synthesis of pectin aerogels or chitosan-coated pectin aerogels. Since curcumin is highly soluble in ethanol (10 mg/mL), the molecules of the drug were easily diffusing inside the gel pore network. Once the curcumin was loaded, the samples were subjected to supercritical drying. Supercritical drying was conducted for 6 h at 120 bar and 40 °C, the conditions optimized for polysaccharides drying [21]. Since curcumin is poorly soluble in supercritical carbon dioxide (2 × 10^−8^ mole fraction for given drying conditions) [22], there was no risk of washing it out during drying.

### 4.3. Characterization

#### 4.3.1. Scanning Electron Microscopy

A scanning electron microscope (Sirion 400 NC, FEI, Hillsboro, OR, USA) was used for determining the surface morphologies of the pectin aerogels and chitosan-coated pectin aerogels. The samples were fractioned and splatter-coated with gold particles prior to the analysis and then scanned at an accelerating voltage of 2–4 kV.

#### 4.3.2. Thermal Analysis

Thermal analysis (thermogravimetry and differential scanning calorimetry) were carried out simultaneously using TGA/DSC1 apparatus (Mettler Toledo, Columbus, OH, USA). Samples were fractioned, weighed (not less than 10 mg) and placed into 100 µL aluminum crucibles. The analyses were performed at an air atmosphere in a temperature range from 30 to 600 °C, with a heating rate of 10 °C per minute.

#### 4.3.3. Textural Properties

Specific surface areas (m^2^/g) of synthetized aerogels were determined by gas N_2_ adsorption-desorption analysis. The experiments were carried out at −196 °C using ASAP 2020MP (Micromeritics Instrument, Norcross, GA, USA). Prior to analysis, the samples were degassed under vacuum at 70 °C for 660 min until obtaining a stable 10 µm Hg pressure. BET (Brunauer–Emmett–Teller) method was employed for determining the specific surface area. Skeletal densities of aerogels were measured by gas pycnometer using AccuPyc II 1340 (Micromeritics Instrument, Norcross, GA, USA), while bulk densities were determined by simply measuring sample mass (weighting) and volume (dimensions). Finally, porosity was determined using Equation (1):

(1) $$Porosity\;\left(\%\right)=1-\;\frac{\rho_{skeletal}}{\rho_{bulk}}$$

where *ρ_skeletal_* is skeletal density, while *ρ_bulk_* is bulk density of aerogels.

#### 4.3.4. Fourier Transform Infrared Spectroscopy

Fourier transform infrared spectroscopy using IRAffinity-1s (Shimadzu, Kyoto, Japan) was employed for characterization of pectin aerogels, chitosan-coated pectin aerogels, and curcumin. The collection of the absorption bands was used to confirm the identity of polysaccharides and curcumin and also for detection of curcumin loaded into aerogels. Aerogels were characterized by ATR-IR method. The samples were cut into halves and placed on the ATR detector. On the other hand, curcumin was ground into a fine powder and dispersed into a matrix made of potassium bromide (KBr). This is the most common method for solids.

### 4.4. Swelling studies

Swelling studies were performed as described elsewhere [6]. Briefly, pectin aerogels and chitosan-coated pectin aerogels (without curcumin) were weighed and immersed in 100 mL of either SGF at pH = 1.2 or SIF at pH = 6.8, conditions mimicking the conditions of the gastrointestinal tract. Samples were collected after selected time intervals, blot-dried with tissue paper for removing excess solution and weighed. All experiments were performed in triplicate.

The swelling ratio was calculated using Equation (2):

(2) $$S_{R}=\frac{M_{S}-M_{0}}{M_{0}}$$

where *M_S_* is the mass of the swollen aerogel after the selected period of time and *M*_0_ is the initial mass of the sample.

### 4.5. In-Vitro Dissolution Tests

In-vitro dissolution tests for pectin aerogels and chitosan-coated pectin aerogels loaded with curcumin were performed using two dissolution media, SGF at pH = 1.2 and SIF at pH = 6.8, as described above. Aerogel samples were firstly placed in SGF for 2 h and immediately transferred to SIF for an additional 22 h.

In-vitro dissolution tests were performed following USP standards [23]. The experiments were carried out at 37 ± 0.5 °C on the Farmatester 3, USP II apparatus (Dema, Ilirska Bistrica, Slovenia). The volume of the dissolution medium was 900 mL, while the speed of rotation was set at 50 rpm. Aliquots of 2 mL for each sample were withdrawn at predetermined time periods and afterwards 2 mL of fresh dissolution medium was added to maintain a constant volume. Samples were subjected to a curcumin assay by a Cary 50 Probe UV spectrophotometer (Agilent Technologies, Santa Clara, CA, USA) at 429 nm. The concentration of curcumin was calculated using the calibration curves in SGF and SIF. All tests were performed in triplicate.

## 5. Conclusions

Two formulations for curcumin were presented in this study, for the purpose of improving the poor bioavailability of the substance. By attaching curcumin to water-soluble polysaccharide aerogels, the dissolution and consequently bioavailability was tremendously improved. While pure curcumin showed almost no dissolution after 24 h, the complete dissolution and release of curcumin from pectin aerogels was achieved after only 3 h and from pectin aerogels coated with an outer layer of chitosan after 24 h. A new coating procedure for pectin aerogels was developed for the purpose of optimizing the release of curcumin (and other active substances as well).

Both formulations proved to be useful for improving the problematic bioavailability of curcumin. On one hand, the pectin aerogels formulation showed burst release. On the other, pectin aerogels coated with chitosan showed controlled release of curcumin over 24 h and maintained a therapeutic dose of the substance.

## Figures and Tables

**Table 1:** Specific surface areas, densities and porosities of pectin aerogels and chitosan-coated pectin aerogels.

	Pectin Aerogels	Chitosan-Coated Pectin Aerogels
Specific surface area, m^2^/g	441 ± 6	276 ± 8
Bulk density, g/cm^3^	0.084	0.098
Skeletal density, g/cm^3^	2.1	1.9
Porosity, %	96.0 ± 0.05	94.8 ± 0.03

**Table 2:** Mass degradation of curcumin powder, pectin powder, and pectin aerogel loaded with curcumin.

	Mass Degradation
Curcumin powder	1st step: 59%
Pectin powder	1st step: 8% 2nd step: 62%
Pectin aerogel loaded with curcumin	1st step: 7% 2nd step: 62%
